# Spectrum of outcomes following traumatic brain injury—relationship between functional impairment and health-related quality of life

**DOI:** 10.1007/s00701-017-3334-6

**Published:** 2017-10-07

**Authors:** Anastasia Tsyben, Mathew Guilfoyle, Ivan Timofeev, Fahim Anwar, Judith Allanson, Joanne Outtrim, David Menon, Peter Hutchinson, Adel Helmy

**Affiliations:** 1 Department of Neurosurgery, Addenbrooke’s Hospital, Hills Road, Cambridge, CB2 0QQ UK; 2 Division of Neurosurgery, Department of Clinical Neurosciences, Cambridge Biomedical Campus, Cambridge, UK; 3 Department of Neurorehabilitation, Addenbrooke’s Hospital, Cambridge, UK; 4 Division of Anaesthesia, Department of Medicine, Cambridge Biomedical Campus, Cambridge, UK

**Keywords:** Traumatic brain injury, Quality of life, SF-36, Glasgow Outcome Scale

## Abstract

**Background:**

The outcome following traumatic brain injury (TBI) is heterogeneous and poorly defined and physical disability scales like the extended Glasgow Outcome Score (GOSE) while providing valuation information in terms of broad categorisation of outcome are unlikely to capture the full spectrum of deficits. Quality of life questionnaires such as SF-36 are emerging as potential tools to help characterise factors important to patients’ recovery. This study assessed the association between physical disability and subjective health rating. The relationship is of value as it may help evaluate the impact of TBI on patients’ lives and facilitate the delivery of appropriate neuro-rehabilitation services.

**Methods:**

A single-centre retrospective study was undertaken to assess the relationship between physical outcome as measured by GOSE and quality of life captured by the SF-36 questionnaire. Cronbach’s alpha was calculated for each of the eight SF-36 domains to measure internal consistency of the test. Multivariate analysis of variance was conducted to look at the association between GOSE and the physical (PCS) and mental (MCS) component scores on the SF-36. Finally, we performed a generalised linear mixed model (GLMM) to assess the relative contribution of GOSE score, age at the time of trauma, sex and TBI duration towards MCS and PCS rating.

**Results:**

There is a statistically significant difference in the MCS and PCS scores based on patients’ GOSE scores. The mean scores of the eight SF-36 domains showed significant association with GOSE. GLMM demonstrated that GOSE was the strongest predictor of PCS and MCS. Age was an important variable in the PCS score while time following trauma was a significant predictor of MCS rating.

**Conclusions:**

This study highlights that patients’ physical outcome following TBI is a strong predictor of the subjective mental and physical health. Nevertheless, there remains tremendous variability in individual SF-36 scores for each GOSE category, highlighting that additional factors play a role in determining quality of life.

## Introduction

Traumatic brain injury (TBI) is a leading cause of mortality and long-term disability among those under 45 and costs an estimated $75 billion each year in the USA alone [9, 12]. Despite improvements in the acute management of TBI, most patients are left with a degree of permanent disability affecting cognitive, psychological and physical function [39]. Commonly used outcome measures such as the functional independence measure (FIM), extended Glasgow Outcome Scale (GOSE) and Disability Rating Scale (DRS) while providing broad categorisation of outcome are limited in their ability to capture the full spectrum of deficits following brain injury [11]. In particular, such scales struggle to measure neurobehavioral disability, which tends to affect a person’s personality, cognition and character [43]. However, it is these aspects that often lead to disintegration of interpersonal relationships, family burden and inability to return to vocation [3, 25].

Quality of life (QoL) questionnaires have gained increasing popularity, allowing for measurements of objective and subjective health indicators. When compared to objective clinical measures of physical function, QoL questionnaires are superior at capturing the patients’ internal judgment of health and factors that may be important to the well being of the individual. This emphasis on Patient Reported Outcome Measures (PROM) is drawing increased interest from both patient groups and funding bodies. Such information in turn may facilitate the delivery and evaluation of neuro-rehabilitation services as well as guide future clinical research. In the TBI population, QoL measures may have the added benefit of capturing the heterogeneity of outcomes and may delineate the natural history of this chronic condition.

The purpose of this study was to investigate the relationship between the functional score of patients with TBI and their subjective health status. The GOSE test was used as a measure of physical function, while the 36-Item Short Form Survery (SF-36) was used as an assessment of QoL. While both these assessment tools have been validated in the TBI population, there has been no definite characterisation of the relationship between physical disability and subjective perception of handicap [18, 24, 27].

## Methods

### Sample and measures

This was a retrospective study of patients seen in Addenbrooke’s Hospital, Cambridge, Neurotrauma Outpatient Clinic between 2005–2013. The majority of individuals underwent treatment for TBI at Addenbrooke’s, with a small proportion of patient’s being referred from other centres. Tests of physical function and subjective health status were administered at each clinic appointment.

The physical disability was assessed using GOSE, an eight-scale global measure of function, which has been validated in TBI [13, 30]. The SF-36 questionnaire was used to assess the patient-reported QoL. The test consists of 36 multiple choice questions that are grouped into eight domains: PF, physical functioning; RP, role limitation due to physical problems; BP, bodily pain; RE, role limitations due to emotional problems; VT, vitality; GH, general health perception; MH, mental health; SF, social functioning. The domain scores were calculated by transforming the raw data into a scale of 0–100 and using Likert’s method of unweighted summed ratings [26, 40]. In the scale, the higher scores indicate better subjective health. Two summary scores, physical component summary (PCS) and mental component summary (MCS), are derived by taking unweighted means of the corresponding domains.

The SF-36 survey was only administered to patients with adequate communication skills who were able to respond to the questionnaire. Thus only patients with GOSE score of 3 or greater were included in the final analysis. Clinic appointments with missing GOSE or SF-36 scores were excluded from analysis. In addition, subjects with incorrect or missing demographic details were also excluded, as these could not be incorporated into the final statistical analysis.

### Statistical analysis

Descriptive statistics were used to summarise the demographic data. Normality of data was tested using the Shapiro-Wilk test while Levene’s and Bartlett’s tests were used to test homogeneity of variance. Based on skewed distribution of the data, non-parametric testing of correlation among MCS, PCS and GOSE were examined using Spearman rank. The level of statistical significance was set at p < 0.05. All analyses were carried out in SPSS 24.0 (IBM SPSS, Chicago, IL, USA).

### Measure of internal consistency of SF-36 domain scores and its association with physical disability

Cronbach’s alpha was calculated for each of the eight domain scores for all patients during their first visit. The minimum threshold for the coefficient was set at 0.7 and preferable above 0.8 [13, 30]. A domain was considered distinct if its respective alpha coefficient exceeded inter-domain correlation of all other scales.

A series of multivariate ANOVAs were conducted with GOSE scores as the independent variable and the two summary scores, MCS and PCS, as the dependent variables. Interaction was also assessed between age of TBI and SF-36 domain scores at the first clinical appointment following discharge.

### Contribution of independent variables to subjective QOL scores

A generalised mixed model (GLMM) was used to assess the relatively contribution of GOSE score, age at the time of trauma, sex and TBI duration on MCS and PCS score. Patient hospital numbers were identified as random effects, while sex, age at the time of trauma, TBI duration and GOSE were fixed effects. GLMM was used to account for the variable time points of questionnaire collections and the unequal sample size observed for each follow-up visit. R^2^s were calculated to determine the goodness of fit of the model.

To look at the general spread of responses on MCS and PCS for each GOSE category, a box and whisker plot was constructed. While there may be a positive relationship between GOSE and SF-36 responses, there may be variability in responses highlighting individual differences in perceived QOL.

### Temporal variation in GOSE and SF-36 parameters

To explore the relative concordance of GOSE and SF36 in identifying changes (improvement or deterioration) in outcome, we identified patients who attended the neurotrauma clinic on at least two occasions. Patients were divided into cohorts in which there was a change in GOSE category and the mean change in SF-36 calculated.

## Results

### Descriptive statistics

A total of 513 adults above the age of 16 who were seen in neurosurgery clinic between 2005–2013 met the inclusion criteria and were included in the final analysis. Table 1 shows the demographic characteristics of the study cohort as well as the disability GOSE score at the first follow-up clinic. Mean age at the time of traumatic brain injury was 39.7 years (range: 16–91 years; SD = 16.9). The range of clinic follow-up dates was 0–611 months, with a total of 922 individual visits collected over this time period. The average number of follow-up clinics for a patient was two, although these were inconsistent as they were based on the clinical need of the individual patients.

**Table 1:** Demographic characteristics of the study population

n	513
Sex	
Female	152 (30%)
Male	361 (70%)
Age at TBI (years)	
Median	38
Mean	39.7
SD	16.9
Range	16–91
GOSE (%) at first follow-up	
Upper good recovery	12.9
Lower good recovery	11.6
Upper moderate disability	30.8
Lower moderate disability	17.9
Upper severe disability	19.1
Lower severe disability	7.7

GOS, Extended Glasgow Scale. SD, standard deviation

### Reliability of SF-36 domain score

Summary scores for the eight domains of SF-36 at the first clinic visit as well as intra-class correlations are summarised in Table 2. The Cronbach’s alpha for the eight SF-36 domains was 0.90 suggesting high internal consistency of the questionnaire. Individual alpha coefficients ranged from 0.87–0.89 and were substantially greater than the correlations between domains. Most domains had a strong inter-item correlation and were worthy of inclusion, resulting in a lower coefficient if deleted.

**Table 2:** Domain characteristics including mean and standard deviation (SD) with reliability statistics: Cronbach’s alpha coefficient and inter-domain correlations

	Domain characteristics		Reliability	
SF-36 domain	Mean	SD	Alpha	Inter-domain correlation mean (range)
PF	67.0	30.9	0.89	0.47 (0.38–0.52)
RP	32.5	41.3	0.89	0.53 (0.43–0.61)
BP	59.7	31.2	0.89	0.54 (0.49–0.59)
GH	58.3	25.0	0.89	0.57 (0.50–0.63)
VI	44.7	25.6	0.88	0.59 (0.43–0.72)
SF	52.7	31.5	0.87	0.62 (0.52–0.71)
RE	44.3	45.5	0.89	0.56 (0.46–0.67)
MH	58.8	24.6	0.88	0.57 (0.38–0.72)

### Association between GOSE and SF-36 scores

Based on the result of the Shapiro-Wilk test of normality, PCS and MCS results were found to be non-parametric (p < 10^−14^). The correlation among PCS, MCS and GOSE was significantly positive, indicating that as the GOSE score of disability improved, the patients’ subjective rating of health also rose. PCS showed the highest positive Spearman rank correlation of 0.67 (p < 0.001) with GOSE. MCS had a correlation of 0.61 (p < 0.001) with GOSE and 0.71 (p < 0.001) with PCS.

**Fig. 1:**
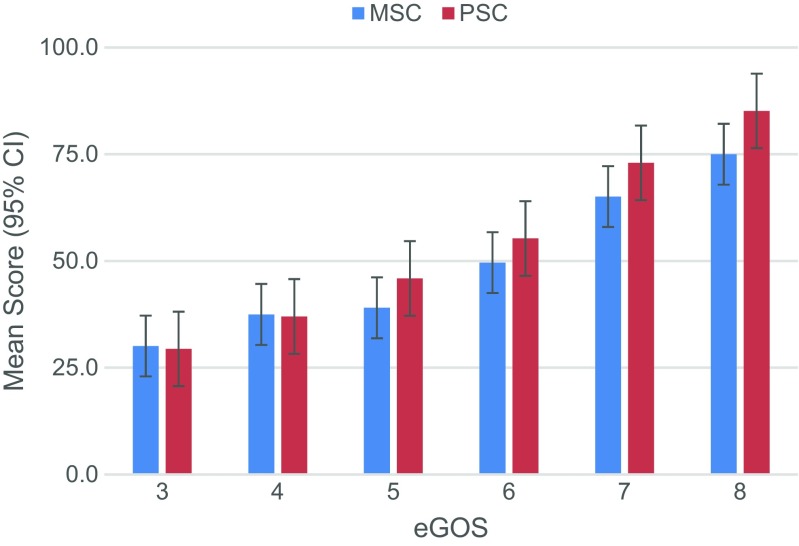
Comparison of GOSE to the two summary scores, PCS and MCS on SF-36. Mean MCS and PCS scores are plotted against GOSE categories (error bars: 95% confidence interval). All summary measures showed increasing scores with more favourable GOSE (multivariate ANOVA all p < 10^−93^) [3=Lower severe disability 4=Upper severe disability 5=Lower moderate disability 6=Upper moderate disability 7=Lower good recovery 8=Upper good recovery]

Analysis using univariate ANOVAs found that GOSE scores had statistically significant effect on both PCS (F = 202.9; p < 0.005; partial η^2^ = 0.53) and MCS (F = 14.9; p < 0.005; partial η^2^=0.41) scores. Likewise, the mean scores of the eight domains of SF-36 also showed a significant association with GOSE scores (Pillai’s trace = 0.68; F = 17.8, p < 0.005; partial η^2^ = 0.14). Comparisons of GOSE and PCS and MCS scores are plotted in Fig. 1.

### Relationship among GOSE, SF-36 domain scores, age at trauma, gender and time since injury

The generalised linear mixed model was used to examine the relationship among GOSE, PCS/MCS, gender, age at the time of trauma and time since injury. The PCS and MCS scores both increased as the GOSE rose. The regression coefficient shows that, all other things equal, for every 1-point rise in GOSE, the score on PCS would rise from 4.1–41.9 points (p < 0.0005). However, relative to GOSE 3, patients with a GOSE 4 score rated 2.6 lower on PCS, although this was not statistically significant (p = 0.266). This suggests a plateau effect at lower GOSE scores, such that physical disability no longer contributes to further declines in GOSE score below 4, while variables contributing to the MCS (which are likely to include cognitive, psychological and psychiatric symptoms) do. In addition, the age at the time of trauma was also statistically significant at p = 0.03, indicating that the older the patient was at the time of TBI, the worse they would score on the PCS domain of SF-36 by approximately 0.07 points per year of age. The clinical significance of this small but statistically significant difference is unclear.

Similarly, the MCS score increased by 8.4–54.7 points for every 1 point rise in GOSE group (p < 0.005). Time since injury showed an increase in MCS score of 0.04 points per year and this was statistically significant at p = 0.018. With a significance value greater than 0.05, there is not enough evidence to conclude whether the gender or progression of time from TBI had an effect on the PCS outcome. Approximately 48% of the variability in the model was explained by the independent variables included in the analysis for PCS and 39% for MCS.

**Fig. 2:**
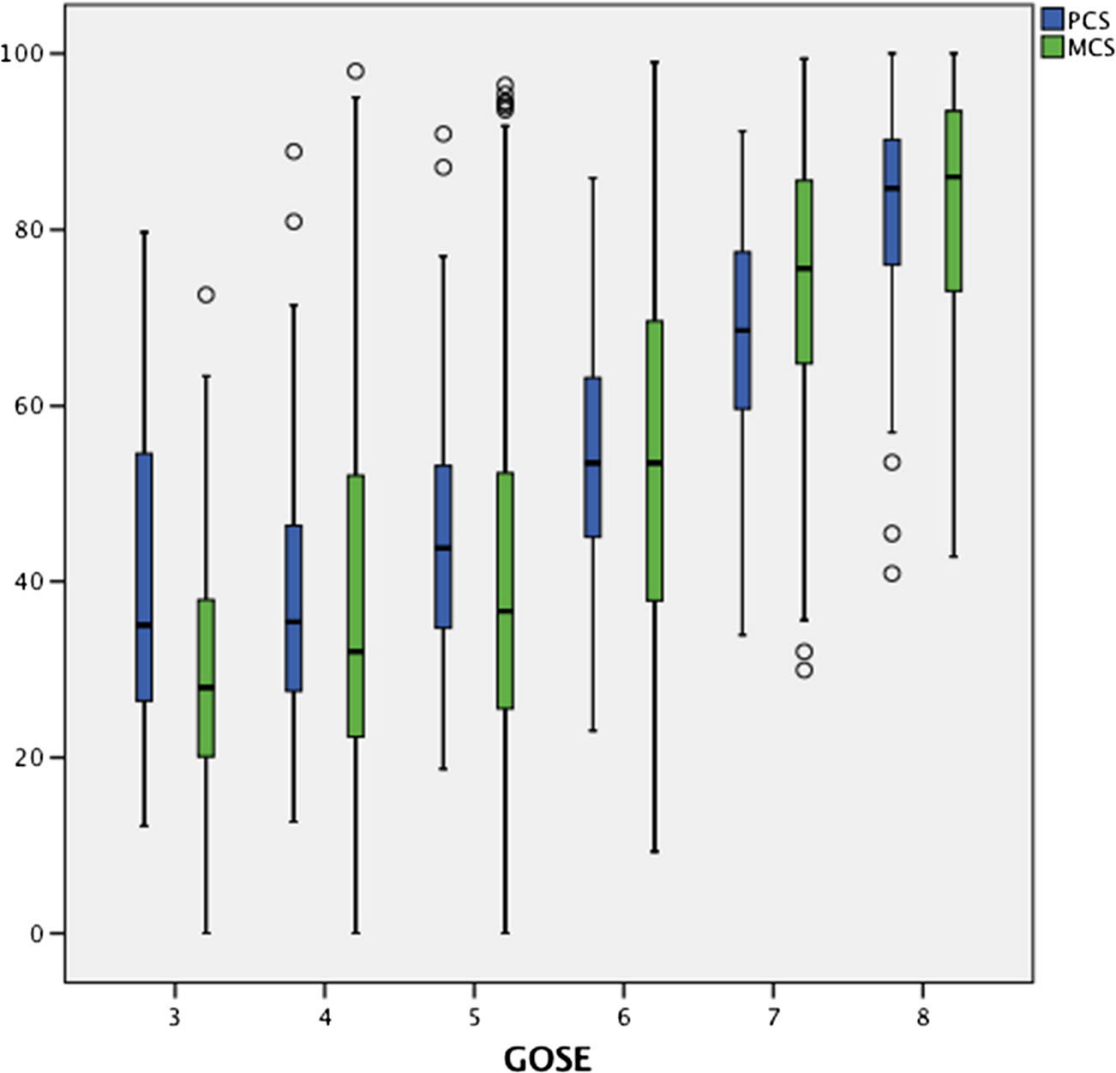
Box and whisker plot showing the spread of MCS and PCS responses for each GOSE category. [3=Lower severe disability 4=Upper severe disability 5=Lower moderate disability 6=Upper moderate disability 7=Lower good recovery 8=Upper good recovery]

Although GOSE had a high explanatory power on PCS and MCS, there was great inter-patient variability of scores as demonstrated in Fig. 2. Within each GOSE score, there is a large spectrum of physical and mental component scores underlining that, on an individual basis, factors other than physical status likely influence patients’ mental and physical health perception. The mean score on MCS was 51.77 (SD 25.60) and PCS 52.46 (SD 19.46) (Table 3).

**Table 3:** Generalised linear mixed model of PCS and MCS scores. SE, standard error

	PCS				MCS			
	β	SE	CI (95%)	p	β	SE	CI (95%)	p
GOSE								
Upper good recovery	41.9	2.5	37.3–46.7	0.000	54.7	2.5	49.8–59.5	0.000
Lower good recovery	27.7	2.5	22.7–32.7	0.000	44.7	3.0	38.9–50.5	0.000
Upper moderate disability	13.2	2.3	8.6–17.8	0.000	25.0	2.6	19.9–30.0	0.000
Lower moderate disability	4.1	2.4	−0.6–8.7	0.049	13.0	2.9	7.3–18.7	0.000
Upper severe disability	−2.6	2.4	−7.3–2.0	0.266	8.4	2.9	2.6–14.2	0.005
Age at trauma	−0.07	0.03	−0.13– (-0.07)	0.03	0.06	0.05	−0.04–0.16	0.24
Gender								
Male	0.10	1.1	−2.1–2.3	0.93	−0.42	2.0	−4.4–3.6	0.84
Months since injury	0.02	0.01	−0.01–0.04	0.08	0.04	0.02	0.01–0.08	0.018
R^2^	0.48				0.39			

**Fig. 3:**
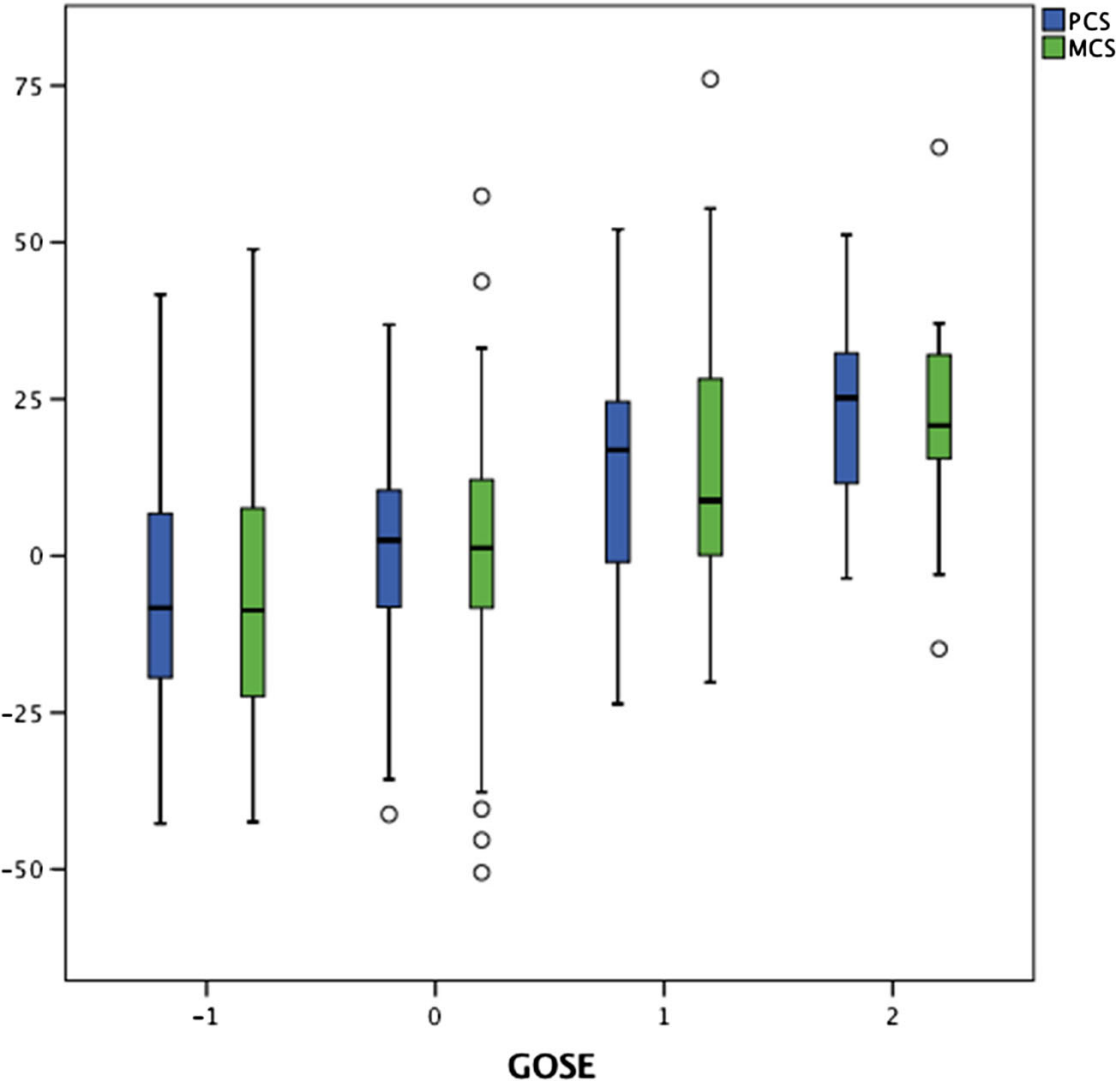
Box and whisker plot showing the change in GOSE score and PCS/MCS score between the 1st and 2nd clinic appointment. Only time points with ten or more subjects are illustrated

A comparison between scores on concurrent clinic visits demonstrated that both PCS and MCS correlated positively with GOSE score. If a patient’s GOSE score dropped during the subsequent visit, their SF-36 score also tended to drop and vice versa (Table 4 and Fig. 3), suggesting that both functional impairment and health-related quality of life metrics change together as patients recover or deteriorate.

**Table 4:** GOSE and PCS/MCS score changes between the 1st and 2nd clinic appointment

GOSE		PCS			MCS		
Score change	Number of subjects	Mean (SD)	Range	Median	Mean (SD)	Range	Median
−4	1	−16.6			−5.8		
−3	1	−31.61			−13.36		
−2	5	−6.68 (21.8)	−31.3–27.6	−6.7	5.28 (19.2)	−14.9–30.8	−0.08
−1	25	−5.25 (20.8)	−42.7–41.7	−8.3	−6.19 (22.5)	−42.4–48.9	−8.7
0	114	0.87 (15.5)	−41.22–36.9	2.52	1.40 (17.9)	−50.5–57.4	1.25
1	46	13.25 (15.6)	−23.6–52.1	16.85	13.31 (20.4)	−20.16–76.02	8.83
2	16	23.85 (16.1)	−3.6–51.2	25.2	22.08 (17.7)	−14.8–65.2	20.78
3	9	19.70 (29.8)	−24.4–59.7	11.47	20.66 (20.8)	−20.1–52.1	21
4	4	24.02 (36.9)	−18.03–66	24.06	22.73 (22.3)	−8.8–41.7	29

SD, standard deviation

## Discussion

The aim of this study was to characterise the relationship between physical outcome following TBI and subjective health scores, as measured by the SF-36 questionnaire. To our knowledge, this is one of the largest cohorts of patients cited in the TBI literature, with a total of 513 patients. The GOSE score had a significant impact on a patient’s PCS and MCS scores and the SF-36 proved to be a robust metric for assessing improvement in GOSE. As the GOSE score increased the subjective rating on MCS and PCS also rose by an average of 33.8 (p < 0.005) points on the MCS and 33.2 (p < 0.005) points on the PCS. These trends are consistent with the well-documented finding that poor physical function and reduced mobility lead to increased pain and decreased independence, as well as precluding return to vocation [22]. The finding of the positive relationship between physical function and QOL is important because it suggests that managing residual physical deficits could lead to an increase in subjective health status. Interestingly, at lower GOSE categories (GOSE 3 and GOSE 4), there appears to be little difference in average PCS scores; however, the MCS continues to decline. Targeted rehabilitation has clearly shown that rehabilitation can decrease disability and improve the quality of living [1, 2, 8, 19]. Even patients with moderate to severe TBI demonstrate some continued neuropsychological recovery several years after injury—particularly in the domain of cognitive speed, visuospatial skills and verbal memory [28].

When combined with age, sex and progression over time since TBI, the model predicted 48% of variability observed in PCS and 39% in MCS scores. Although the physical function played a critical role in QOL rating, it was not the sole determinate and other factors not included in the model influence subjective health. This is in line with Ruff and colleagues who found that social function and return to vocation depended more on factors such as neuropsychological function rather than physical disability [32].

The relationship between SF-36 and GOSE is consistent with findings reported by Wilson et al., who found a positive correlation between GOSE and eight subscales of SF-36, particularly in the social functioning domain [42]. A meta-analysis of 49 studies found SF-36 to be the most widely used and robust tool for assessing outcome following TBI, with strong internal consistency and interpretability [31]. However, as mentioned by the authors, SF-36 may not be a sensitive tool for detecting emotional and cognitive disturbances. In addition to the previous reported research, this study highlights that GOSE is the strongest predictor of PCS and MCS scores. There is also a statistically significant difference in the MCS and PCS scores based on patients’ GOSE scores.

Our cohort of patients had higher average PCS scores (M = 52.46, SD ± 19.46) compared to MCS (M = 51.77, SD ± 25.60). This is in agreement with results of the study by Steinbuechel et al., Hawthorne et al. and Wilson et al. [20, 36, 41]. In contrast, other studies found reverse results with subjects having higher MCS than PCS [15, 17, 21, 34]. They argued that the discrepancy between the mental and physical scores could result from lack of awareness in patients with severe TBI. However, in the study by Steinbuechel et al. the number of patients with severe TBI was double of those with mild TBI. In terms of distribution of GOSE scores, we had a relatively equal number of subjects with GOSE below and above a score of 5.

Age at the time of trauma was a positive predictor of PCS score but the effect size was small. For every additional year, the PCS score decreased by 0.07 points (p < 0.05). On the other hand the MCS score tended to rise with increasing age, although this finding was insignificant (B = 0.04, p = 0.36). This is in accordance with other studies, which found that the proportion of poor outcome following TBI increased with age [16, 38]. Despite the changes observed in the ageing brain, including modification in electrical coupling and cell connections, it retains remarkable ability to respond to stimulation and improve working memory [5]. For instance, targeted rehabilitation programmes have been found to increase memory in patients with mild to moderate Alzheimer’s disease [10] and focused training of motor skills in Parkinson’s patients lead to increased activity within motor regions of the brain [37]. These studies provide compelling evidence that targeted therapy and rehabilitation in TBI patients, despite their age, may yield positive benefits on the cognitive function. These in turn may facilitate patients’ return to vocation and improving their emotional health. Nevertheless, there are additional challenges following trauma in the elderly associated with increased co-morbidities and not only with neuronal changes [6]. As such, this group of individuals may require additional in-hospital support and physiotherapy to promote their return to the community.

Despite the positive relationship between GOSE and SF-36 domain scores, there is tremendous variability in MCS and PCS scores within each of the GOSE categories. This is consistent with the findings of Polinder et al., whose meta-analysis found significant heterogeneity in SF-36 summary scores [31]. GOSE is a global score, which captures only a limited component of a subject’s overall health. Factors such as degree of cognitive ability, educational background and psychological health have all been found to affect QOL post TBI. On the other hand, the majority of studies have found no gender differences in physical and cognitive function following discharge [4, 29, 33, 35]. This is in keeping with our results that show gender to be a non-significant variable in influencing SF-36 rating. Interpersonal differences in social and economic status following trauma may also be an important contributing factor to outcome. For instance, economic advantage can allow better provision of home support, both physical and mental. It could assist patients in paying for private therapies to enhance psychological and physical health. In addition, the family network and meaningful interpersonal relationship play a crucial part in the support and rehabilitation of patients following TBI.

This study has several limitations. First, it is a retrospective single-centre study from a specialised tertiary neurotrauma clinic in which the population demographics, socioeconomic background and availability of rehabilitation services may be skewed and may not necessarily reflect the UK general TBI population. In addition, there were a limited number of fixed effects included in the study. Factors such as education, employment status, relationship status and cultural background were not included in the statistical analysis because of incomplete information. These factors have been found to predict outcome following TBI and may explain some of the variability seen in the scores of MCS and PCS observed for our cohort. For example, research has shown that pre-injury employment status and educational level are strong predictors of QOL and return to vocation [23]. Likewise, cognitive ability has been shown to account for 21% to 30% of the variability observed in the functional ability post trauma [7].

Our patients were not subdivided based on the severity of the trauma. As mentioned earlier, some studies have found that subjects with severe TBI lacked awareness and thus ranked higher on QOL questionnaires. Self-awareness is directly associated with motivation and change of behavior, which may facilitate better functional outcomes following trauma. On the other hand, it is also more likely to result in depression and other psychological conditions further decreasing perceived QOL [14].

## Conclusion

The aftermath of TBI is heterogeneous, leaving patients with a spectrum of physical, cognitive and psychological sequelae. This study highlights that patients’ physical outcome following TBI is a strong predictor of their subjective mental and physical health. Nevertheless, this is not the only factor to predict QOL post trauma, as there is a tremendous variation in scores observed across the GOSE spectrum. Thus, despite poor physical function, some patients rate their quality of life as high and vice versa. Further analysis of data would include additional factors such education, employment status, family network and cognitive ability to better model the predictors and help understand the natural history of recovering following TBI. This in turn could facilitate delivery of appropriate and individualised rehabilitation to ensure the best possible outcome for patients with TBI.

## Abbreviations

DRS: Disability Rating Scale
FIM: Functional independence measure
GLMM: Generalised linear mixed model
GOSE: Extended Glasgow Outcome Score
MCS: Mental Component Score (SF-36)
PCS: Physical Component Score (SF-36)
PROM: Patient Reported Outcome Measures
QoL: Quality of life
SF-36: 36-Item Short Form Survery
TBI: Traumatic brain injury
